# The safety and efficacy of ERCP in the pediatric population with standard scopes: Does size really matter?

**DOI:** 10.1186/s40064-016-1749-9

**Published:** 2016-02-19

**Authors:** Abdullah Emre Yıldırım, Reskan Altun, Serkan Ocal, Murat Kormaz, Figen Ozcay, Haldun Selcuk

**Affiliations:** Department of Gastroenterology, Faculty of Medicine, Başkent University, 06500 Bahcelievler, Ankara, Turkey; Department of Pediatric Gastroenterology, Faculty of Medicine, Başkent University, Ankara, Turkey

**Keywords:** Endoscopic retrograde cholangiopancreatography, Childhood, Duodenoscope, Pediatric ERCP

## Abstract

Experience with endoscopic retrograde cholangiopancreatography in the pediatric population is limited. The aim of this study was to evaluate the outcomes of ERCP in the pediatric population performed by adult gastroenterologists with standard duodenoscopes. This study is a structured retrospective review of endoscopic reports, computerized and paper medical records, and radiographic images of patients under the age of 18 who underwent ERCP for any indication at a tertiary referral centre. Data regarding demographic characteristics and medical history of patients, indications, technical success rate, final clinical diagnosis, and complications were analyzed. Forty-eight children with a mean age of 13 years (range 2–17) underwent a total of 65 ERCPs. The indications of ERCP were as follows; suspected choledocholithiasis (55 %), post-liver transplantation anastomotic biliary strictures (21 %), post-surgical bile duct injury (10 %), choledochal cyst (2 %), recurrent or chronic pancreatitis (10 %), and trauma (2 %). The cannulation success rate in the overall procedure was 93.8 %. Therapeutic interventions were performed in 70.7 % of patients. Post ERCP pancreatitis was the most common complication occurring in 9.2 % of patients, and no procedure related mortality occurred. When performed by well-trained adult gastroenterologists, the use of endoscopic retrograde cholangiopancreatography with standard duodenoscopes is safe in pediatric population.

## Background

Endoscopic retrograde cholangiopancreatography (ERCP) has long been established as an effective diagnostic and therapeutic modality for pancreaticobiliary disorders in the adult population (Adler et al. 2005). In Waye 1976 reported the first successful ERCP in a 3.5-month-old child with cholestasis using a standard duodenoscope (Waye 1976). The use of ERCP in pediatric population was limited; however, since the development of smaller diameter duodenoscopes and accessories in late 80 s, its use has grown considerably (Allendorph et al. 1987; Fox et al. 2000). Experience was limited due to multiple factors, including the relatively low incidence of diseases requiring ERCP in this age group and the impression that the procedure is technically difficult in children. Additionally, the indications and safety of ERCP in children have not been well defined (Hsu et al. 2000).

The aim of the present study was to evaluate the indications, results, and safety of ERCP in pediatric population performed by adult gastroenterologists with standard duodenoscopes.

## Methods

This study is a structured retrospective review of medical records of patients under the age of 18 who underwent ERCP in Baskent University Department of Gastroenterology between 2001 and 2012. This chart and computer-based study was approved by the Baskent University Institutional Review Board.

All procedures were performed by the same gastroenterologists with appropriate training and expertise in ERCP. Procedures were done at the Baskent University Ankara Hospital, a tertiary centre with expertise in solid organ transplantation and hepatobiliary surgery. Informed written consent was obtained from the legal guardian of the patients. All patients were placed under deep sedation by an anesthesiologist and the procedures were performed in the prone position.

For all patients, a diagnostic adult duodenoscope (TJF-160; Olympus America Corp., Melville, NY, USA) with an insertion diameter of 10.8 mm or a therapeutic duodenoscope (TJF-100; Olympus America Corp., Melville, NY, USA) with an insertion diameter of 12.5 mm was used at the discretion of the gastroenterologist. Technical success was defined as a successful deep cannulation of the bile duct or pancreatic duct along with completion of any planned diagnostic or therapeutic procedure. Cannulation was achieved using a tapered tip cannulation catheter with a guidewire. Sphincterotomy was performed in standard fashion. Stone removal was achieved using the passage of Fogarty-type balloon. Ductal clearance was documented with balloon occlusion cholangiogram. For patients in whom ductal clearance was not achieved, a plastic biliary stent was placed in the standard fashion.

Complications were defined as occurring within 2 weeks of the procedure and were further categorized as bleeding (in which endoscopic therapy and/or blood transfusion was required), perforation, post-ERCP pancreatitis, cholangitis, abdominal pain in the absence of cholangitis and/or pancreatitis, and death.

Data regarding demographic characteristics and medical history of patients, indications, technical success rate, final clinical diagnosis, ERCP interventions performed, and complications were obtained from the hospital electronic medical records database and retrospectively analyzed.

IBM SPSS Statistics 19 (SPSS, Inc., Chicago, IL, USA) program was used for statistical analysis. Results were presented as median (minimum–maximum) and percentages.

## Results

Over the 10-year study period, 65 ERCPs were performed, including 33 (51 %) diagnostic and 32 (49 %) therapeutic procedures on 48 children at our institution. There were 20 (42 %) girls and 28 (58 %) boys aged from 2 to 17 years. The median age was 13 years (2–17 years). Also, 42 % (n = 20) were pre-adolescence and 48 % (n = 28) were teenagers. The majority of the patients (n = 37) underwent a single procedure, 7 underwent two procedures, 3 underwent three procedures and 1 underwent five procedures (Table 1).

**Table 1:** Patients’ demographics

Gender (%)	
Female	20 (42 %)
Male	28 (58 %)
Median age (max–min)	13 (2–17)
ERCP procedures (%)	
Diagnostic	33 (51 %)
Therapeutic	32 (49 %)

Of all procedures, 91 % were for biliary and 9 % for pancreatic indications. The biliary indications were suspected choledocholithiasis (55 %), post liver transplantation anastomotic biliary strictures (21 %), post-surgical bile duct injury (10 %), and choledochal cyst (2 %). The pancreatic indications included recurrent or chronic pancreatitis (10 %) and trauma (2 %). The final diagnoses after the procedure are demonstrated in Table 2. A normal cholangiography was found in 22.9 % of ERCPs.

**Table 2:** Final diagnosis after ERCP

	n (%)
Choledocholithiasis	12 (25 %)
Post-liver transplantation biliary strictures	11 (22.9 %)
Normal	11 (22.9 %)
Post-surgical bile duct injury	3 (6.2 %)
Biliary strictures	2 (4.1 %)
Pancreas divisium	2 (4.1 %)
Choledochal cyst	1 (2.1 %)
Chronic pancreatitis	1 (2.1 %)
Pancreatic duct injury	1 (2.1 %)
Unsuccessful ERCP	4 (8.4 %)

An overall procedure, papilla cannulation success rate was 93.8 %. Cannulation success rate per patient was 91.7 % (44 of 48 patients). Of these 44 patients, 93.2 % resulted in successful cannulation in first ERCP procedure and 6.8 % in second procedure. Additionally, successful cannulation was achieved 88.6 % (39 of 44 patients) with standard cannulation techniques. In five patients (11.4 %), pre-cut sphincterotomy (with needle-knife in 1 patient and standard sphincterotome in 4 patients) was performed when standard techniques fail to achieve cannulation (Table 3).

**Table 3:** ERCPs success, cannulation properties and techniques rates

	N	%
Overall ERCP procedures, papilla cannulation success	61	93.8 (61/65)
Cannulation success rate per patients	44	91.7 (44/48)
Successful cannulation in first ERCP procedure	41	93.2 (41/44)
Successful cannulation in second ERCP procedure	3	6.8 (3/44)
Successful cannulation with standard cannulation techniques	39	88.6 (39/44)
Successful cannulation with precut sphincterotomy techniques	5	11.4 (5/44)

Therapeutic interventions were performed in 70.7 % of patients including sphincterotomy in 38 patients (58.5 %), balloon sweep in 17 patients (26.1 %), biliary plastic stent insertion 12 patients (18.5 %), stone extraction in 8 patients (12.3 %), nasobiliary drainage tube placement in 6 patients (9.2 %), and balloon dilatation of biliary strictures in 1 patient (1.5 %) (Table 4).

**Table 4:** Therapeutic interventions (65 procedures in 48 patients)

	n (%)
Sphincterotomy	38 (58.5 %)
Balloon sweep	17 (26.1 %)
Biliary plastic stent insertion	12 (18.5 %)
Stone extraction	8 (12.3 %)
Nasobiliary drainage tube placement	6 (9.2 %)
Ballon dilatation of biliary strictures	1 (1.5 %)

The complication rate was 12.3 % (8 of 65 procedures) per procedure and 16.6 % (8 of 48 patients) per patients. Complications are summarized in Table 5. Post ERCP pancreatitis was the most common complication, with an occurrence rate of in 9.2 %. All six patients were graded as mild pancreatitis according to the consensus classification of post-ERCP pancreatitis by Cotton et al. (1991), and all resolved with conservative treatment. Bleeding occurred in 2 patients (3.1 %) and controlled with endoscopic management. Delayed hemorrhage did not occur. No severe pancreatitis, bleeding, perforation and infection occurred. There was no procedure related mortality.

**Table 5:** Complications related to ERCP

	N	% of total ERCP
Post ERCP pancreatits	6	9.2
Bleeding after sphincterotomy	2	3.1
Total	8	12.3

## Discussion

In present study, the rate of technical success and the rate of complications found in pediatric ERCP were in concordance with previously reported large series (Cheng et al. 2005; Giefer and Kozarek 2015; Enestvedt et al. 2013; Troendle and Barth 2013, 2015; Halvorson et al. 2013; Otto et al. 2011; Jang et al. 2010; Iqbal et al. 2009) (Table 6). We suggest that ERCP is a safe and effective method for both the diagnosis and treatment of pediatric patients.

**Table 6:** Pediatric endoscopic retrograd cholangiopancreatography larger series

References	Mean age (range)	Study design	Endoscopist	Patient (n)	ERCP (n)	Procedure success (%)	Therapeutic (%)
Giefer and Kozarek (2015)	13.6 years (2 months–18 years)	Retrospective	Adult	276	425	95	81
Troendle et al. (2015)	12.7 years (1 months–19 years)	Retrospective	Pediatric and adult	313	432	NR	86
Enestvedt et al. (2013)	14.9 years (3 months–21 years)	Retrospective	Adult	296	429	95	64
Halvorson et al. (2013)	12 years (6 years–17 years)	Retrospective	Adult	45	70	99	93
Troendle and Barth (2013)	15.2 years (1 months–18.4 years)	Retrospective	Pediatric	65	154	100	100
Otto et al. (2011)	11.4 years (2 months–21 years)	Retrospective	Adult	167	231	NR	69
Jang et al. (2010)	8 years (1 months–16 years)	Retrospective	Pediatric	122	245	98	78
Iqbal et al. (2009)	NR	Retrospective	Adult	224	343	NR	43
Cheng et al. (2005)	9.3 years (1 months–17 years)	Retrospective	Adult	245	329	98	71

*NR* not specifically reported

When performed by experienced endoscopists, ERCP has an acceptable complication rate in the adult population (Rabenstein et al. 1999). In the pediatric population, experience with ERCP has been limited due to multiple factors including a relatively low incidence of diseases requiring ERCP and the impression that the procedure is technically difficult in children. Additionally, the indications and safety of ERCP in the pediatric population have not been well defined. Few pediatric gastroenterologists receive sufficient training on advanced endoscopy including procedures such as ERCP. This gap is generally filled by adult gastroenterologists.

The main problem is that most adult endoscopy units do not have pediatric duodenoscopes. Pediatric duodenoscopes are recommended for children <10 kg or younger than 12 months of age, beyond which an adult diagnostic or therapeutic duodenoscope is acceptable (ASGE Technology Committee et al. 2012). However, a diagnostic duodenoscope or a therapeutic duodenoscope was used at the discretion of the gastroenterologist in this study, and no complications such as tracheal impressions, airway obstruction, nor the necessity of endotracheal intubation occurred.

In Western countries, the most common indications for ERCP are choledocholithiasis and acute or chronic pancreatitis whereas the most frequent indication in Eastern countries (Japan, Korea and India) is congenital choledochal cysts (Cheng et al. 2005; Pfau et al. 2002; Teng et al. 2000; Issa et al. 2007; Jang et al. 2010; Otto et al. 2011). The most common indication in this study was choledocholithiasis. Additionally because of Baskent University hospital is a tertiary centre with expertise in solid organ transplantation, post-liver transplantation biliary strictures had a similar frequency. Enestvedt et al. reported their pediatric ERCP experience in 296 children who underwent a total of 429 ERCPs. The most common indications in this series were similar to present study such as choledocholithiasis (26.1 %) and biliary stricture (26.1 %) (Enestvedt et al. 2013).

Conscious or deep sedation and topical anesthesia has become a safe alternative to general anesthesia in pediatric endoscopy (Green et al. 2001). But the higher complexity and duration of the procedure in pediatric patients with a smaller anatomy sometimes requires general anesthesia. Although the many therapeutic interventions were applied to our pediatric patients and the youngest patient was 2 years old, none of our patients required general anesthesia. Conscious or deep sedation were preferred for all patients unlike other pediatric series (Cheng et al. 2005; Paris et al. 2010; Halvorson et al. 2013; Troendle and Barth 2013). There were also no serious adverse events such as cardiorespiratory suppression specifically related to deep sedation. These findings suggest that the preference between deep sedation and general anesthesia can be determined according to many factors including patient age, weight, and experience.

The most difficult aspect of the ERCP is the first step: selective biliary cannulation (SBC) which may often end in failure. The principles of SBC in children are similar to those used in adult patients. There are some limitations in pediatric patients such as phenotypic characteristics of age range and equipment sizes. Despite all disadvantages, the rate of successful cannulation is as good as comparable to reports in adults (Allendorph et al. 1987; Guelrud et al. 1994; Otto et al. 2011). Success rates of pediatric ERCP were reported 89.5–100 % in previous studies (Teng et al. 2000; Paris et al. 2010; Green et al. 2007; Cheng et al. 2005; Giefer and Kozarek 2015; Enestvedt et al. 2013). The success rate in this study is similar to those in previous reports. Cannulation was unsuccessful in four patients, all of whom were younger than five. The diameter of the ERCP catheter and the sphincterotome is larger than the diameter of the papilla. In our experience, this diameter mismatch increases the difficulty of achieving selective bile duct cannulation in small children and infants.

There were a remarkable amount of therapeutic interventions (70.7 %) in this pediatric patient series. In most of the procedures, more than one intervention was performed including sphincterotomy with balloon sweep or sphincterotomy or balloon sweep with subsequent biliary plastic stent insertion.

The overall complication rate found in the present study (12.3 %) reflected the findings found in literature with regard to adult and pediatric patients (1 %-17,5 %) (Allendorph et al. 1987; Cotton et al. 1991; Cheng et al. 2005; Pfau et al. 2002; Teng et al. 2000; Paris et al. 2010; Halvorson et al. 2013; Enestvedt et al. 2013). Post-ERCP pancreatitis, the most common ERCP-related complication in children, was found to occur in 9.2 % of total ERCP in this study, which is similar to the incidence typically reported in early series (3–17 %) (Fox et al. 2000). Cheng et al. reported that the rate of pancreatitis after therapeutic ERCP was higher than diagnostic ERCP (11.1 vs 5.4 %, respectively) (Cheng et al. 2005). The occurrence of pancreatitis in our patient population may be associated with the high rate of therapeutic interventions. The reported incidence of other complications are as follows; hemorrhage 1–4 %, perforation 1–2 %, and cholangitis 1–5 % (Freeman et al. 1996; Masci et al. 2001; Feurer and Adler 2012; Vandervoort et al. 2002; Wang et al. 2009). Post-ERCP complication rates vary widely depending on the complexity of the intervention.

This study has several limitations that should be noted. First, it is retrospective nature. Complete medical reports such as weight and BMI percentile of the pediatric patients were not available on all patients. Because of the fact that our facility was a referral centre, followup was shorter than 30 days. Short follow-ups consequently led to limited information retrieval on the effect of post ERCP in survival, late complications, and recurrence of the presenting disease. It is a single-center study from a tertiary referral centre. Therefore, the outcomes of this study cannot be generalized as these factors can vary based on local referral patterns, pre/peri/post-procedural medical care, and endoscopist experience.

Based on the results of our study, we suggest that diagnostic and therapeutic ERCP is safe and effective in the pediatric population when performed by an experienced adult gastroenterologist at high-volume centers. The complication rates are comparable to those that observed in adults.
